# Effects of ATP on the Physicochemical Properties and Cytocompatibility of Calcium Sulfate/Calcium Citrate Composite Cement

**DOI:** 10.3390/ma16113947

**Published:** 2023-05-25

**Authors:** Xiangyue Liu, Hong Chen, Haohao Ren, Bo Wang, Xiaodan Li, Suping Peng, Qiyi Zhang, Yonggang Yan

**Affiliations:** 1 College of Physics, Sichuan University, Chengdu 610065, China; 2020222020010@stu.scu.edu.cn (X.L.);; 2 School of Chemical Engineering, Sichuan University, Chengdu 610065, China

**Keywords:** ATP, calcium sulfate hemihydrate, calcium citrate tetrahydrate, composite bone cement, cytoactivity

## Abstract

Adenosine triphosphate (ATP), acting as a source of energy, has effects on cellular activities, such as adhesion, proliferation, and differentiation. In this study, ATP-loaded calcium sulfate hemihydrate/calcium citrate tetrahydrate cement (ATP/CSH/CCT) was successfully prepared for the first time. The effect of different contents of ATP on the structure and physicochemical properties of ATP/CSH/CCT was also studied in detail. The results indicated that incorporating ATP into the cement did not significantly alter their structures. However, the addition ratio of ATP directly impacted the mechanical properties and in vitro degradation properties of the composite bone cement. The compressive strength of ATP/CSH/CCT gradually decreased with an increasing ATP content. The degradation rate of ATP/CSH/CCT did not significantly change at low concentrations of ATP, but it increased with a higher ATP content. The composite cement induced the deposition of a Ca-P layer in a phosphate buffer solution (PBS, pH = 7.4). Additionally, the release of ATP from the composite cement was controlled. The ATP was controlled releasing at the 0.5% and 1% ATP in cement by the diffusion of ATP and the degradation of the cement, whereas it was controlled by the diffusion process merely at the 0.1% ATP in cement. Furthermore, ATP/CSH/CCT demonstrated good cytoactivity with the addition of ATP and is expected to be used for the repair and regeneration of bone tissue.

## 1. Introduction

Bone is an essential tissue for humans to store and metabolize required minerals and supports the human body in performing various movements [1,2]. Small-sized bone defects can heal by themselves; however, supercritical area bone defects induced by osteoporosis, serious trauma, and tumor resection require a clinical operation, as they cannot self-repair [3]. Therefore, bone implant materials for clinical operations have received considerable attention [4,5,6]. Bone cement, a biomaterial that can be arbitrarily shaped and quickly hardens for bone repair, can replace natural bone by filling irregular bone defects [7,8,9].

Ideal bone cement should have suitable degradation rates so that it can be gradually replaced by new bone during the degradation process [10]. For decades, calcium sulfate dihydrate (CSD) cement has been a promising candidate for developing biodegradable bone repair materials because of its self-setting property, desirable biocompatibility, and good osteoconductivity [11]. However, CSD cement degrades faster than new bone forms, which limits its application [12]. To obtain suitable biodegradation rates for CSD cement, one of the most effective strategies is to add slow-biodegradable biomaterials into CSD cement to form composite cement. Xu et al. incorporated calcium phosphate and silk fibroin nanofiber into CSD cement [13]. Although the degradation rate of this composite bone cement was slower than that of traditional CSD cement, its weight loss rate still reached 55.5% after 28 days of in vitro degradation. Huan et al. incorporated tricalcium silicate into CSD cement to adjust the degradation rate of the bone cement [14]. However, the compressive strength of the composite bone cement was below 4 MPa. Recently, Chen et al. successfully fabricated composite bone cement with a suitable degradation rate in vitro by incorporating calcium citrate tetrahydrate (CCT) into traditional CSD cement [15]. This CCT-containing cement showed high mechanical strength. Because C_6_H_5_O_7_^3−^ has a strong absorbability towards Ca^2+^, it partially chelates with the Ca^2+^ in CSD, resulting in strong interfacial interactions between CCT and CSD [16,17]. These strong interfacial interactions significantly improve the mechanical strength of the composite cement. Moreover, the citrate ions, which can be released from CCT, are conducive to the formation of hydroxyapatite (HA) in vivo [18,19,20]. Furthermore, a previous study with Japanese white rabbits concluded that CCT could positively stimulate fracture healing in the early stages when defects were not yet too severe [21,22]. Thus, the combination of both CSD and CCT is an effective strategy for developing bone repair materials with good therapeutic properties.

Adenosine triphosphate (ATP), a high-energy compound, is crucial for biological activities as an energy transfer molecule, a phosphate donor, and a signaling molecule in various living cells [23,24,25]. Studies have shown that ATP can induce the differentiation of bone mesenchymal stem cells (BMSCs) into osteoblasts [26,27]. Inducing the differentiation of BMSCs into osteoblasts can be used to treat osteoporosis, a systemic disorder of bone metabolism [28,29]. With an increasingly aging population, osteoporosis has dramatically risen and poses a significant problem worldwide. Owing to a decline in bone density and bone quality, the patients are susceptible to pain in the back and bones and even fractures, which brings heavy psychological pressure and economic burdens to them and their families [30,31,32]. In the event of a serious fracture, filling bone cement into the bone defect is an effective surgical method [33,34]. Therefore, research on functional biodegradable cement for osteoporosis treatment has become essential. Incorporating chemical medicines that can differentiate BMSCs into osteoblasts may be an effective strategy for obtaining composite cement for osteoporosis treatment.

Chitosan and its derivatives are useful natural bipolymers in the biomedical area due to their biocompatibility, biodegradability, and stimulatory effect on bone formation [35,36,37]. Moreover, Lin et al. successfully developed anti-washout tricalcium silicate bone cement by the addition of carboxymethyl chitosan (CMCS) into the hydration liquid [38]. With the inspiration from anti-washout tricalcium silicate bone cement, CMCS was introduced into the hydration liquid to improve the anti-washout ability of the bone cement. Furthermore, as ATP is ternary acid, calcium hydroxide (CH) was introduced into the liquid phase to weaken the acidity of the hydration liquid by acid-base neutralization reaction.

In this study, ATP was successfully introduced into a bone cement system composed of calcium sulfate hemihydrate/calcium citrate tetrahydrate cement (ATP/CSH/CCT) for the first time. Furthermore, CMCS and CH solutions were introduced into the hydration liquid to improve the anti-washout ability of the cement and to weaken the acidity of the hydration liquid, respectively. The chemical structures, compressive strengths, in vitro degradability, and drug release kinetics of the cement were investigated. As the aim of introducing ATP into bone cement is to improve the cytoactivity of the composite bone cement, the cytocompatibility of ATP/CSH/CCT was studied in detail.

## 2. Materials and Methods

### 2.1. Materials

Calcium citrate (Ca_3_(C_6_H_5_O_7_)_2_; CC), carboxymethyl chitosan (carboxylation degree ≥80%; CMCS), and adenosine triphosphate (C_10_H_16_N_5_O_13_P_3_; ATP) were purchased from Macklin Biochemical Technology (Shanghai, China). Calcium sulfate dihydrate (CaSO_4_·2H_2_O; CSD) was purchased from Sigma-Aldrich (St. Louis, MO, USA), and calcium hydroxide (Ca(OH)_2_; CH) was purchased from Kelong Chemical Co., Ltd. (Chengdu, China).

### 2.2. Preparation and Characterisation of ATP/CSH/CCT

Calcium sulfate hemihydrate (CaSO_4_·1/2H_2_O; CSH) powder was obtained by the hydrothermal method [39]. The CSH powder was obtained by heating reagent-grade CSD in an autoclave (YM30Z, Shanghai Sanshen, Shanghai, China) at 120 °C for 8 h.

**Figure 1:**
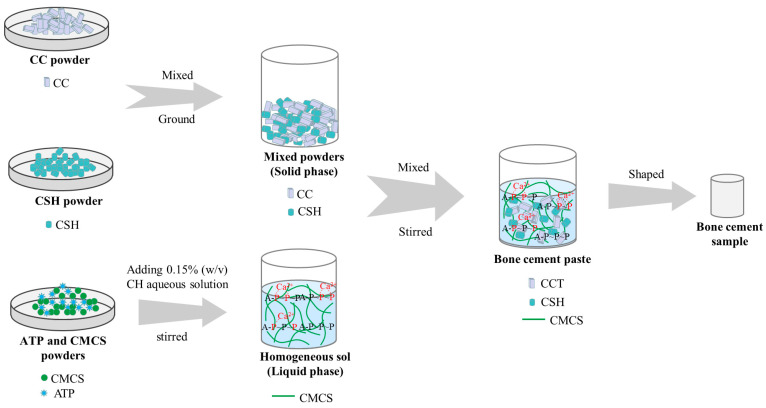
Schematic diagram of the preparation of bone cement samples.

The solid phase of the composite cement was composed of CSH and CC, whereas the liquid phase was a sol formed by ATP, CMCS, and 0.15% (*w*/*v*) CH in an aqueous solution. The compositions of the solid and liquid phases are listed in Table 1. To obtain the solid powders, the CSH and CC powders were premixed and ground by a ball mill at 20 r/min for 6 h. The solid powders were thoroughly mixed with the solution at a liquid-to-solid ratio of 0.3 mL/g and then stirred to form a paste, which was then placed in a polytetrafluoroethylene mold to obtain cylindrical samples of cement. The sample sizes were ɸ 6 × 7 mm or ɸ 6 × 12 mm for different testing. After curing, the cements were removed from the mold for further testing. The preparation process of the composite cement samples is shown in Figure 1.

The composition and structure of the samples were analyzed using X-ray diffraction (XRD; Empyrean, Malvern Panalytical, Dordrecht, The Netherlands) and Fourier transform infrared (FT-IR) spectroscopy (Nicolet 6700, Thermo Fisher Scientific, Waltham, MA, USA). The surface morphology of the cement was examined using scanning electron microscopy (SEM; JSM-5600LV, JEOL, Tokyo, Japan) at an accelerating voltage of 15 kV. The main elements on the cement surfaces were examined using energy-dispersive X-ray spectroscopy (EDS; X-Max, Oxford Instrument, Oxford, UK). To evaluate the densities and diameter shrinkage ratios of samples, the ATP/CSH/CCT cement with a size of ɸ 6 × 7 mm was cured in the air for three days. The densities of samples were calculated through mass divided by volume. Additionally, the diameter shrinkage ratios of the sample were calculated by the change in diameter before and after curing for three days.

### 2.3. Preparation and Characterisation of CH-ATP

A total of 15 mL of the 0.15% (*w*/*v*) CH aqueous solution was mixed with 1 mL of the 10% (*w*/*v*) ATP aqueous solution to obtain a suspension to explore the form and function of ATP in bone cement. Then, the suspension was centrifuged (10,000 rpm, 5 min), and the resulting sediment was dried at 35 °C for 24 h in a vacuum drying oven and labeled as CH-ATP. The prepared CH-ATP was characterized by XRD and FT-IR to investigate the coordination sites of Ca^2+^ with ATP.

### 2.4. Compressive Strength

The compressive strength of the cement was measured using a mechanical testing machine (Instron 5567, Instron Corporation, Canton, MA, USA) at a 1 mm/min loading rate. The sample size was ɸ 6 × 12 mm and six replicates were performed for each group [40].

### 2.5. Weight Loss and pH Value

To evaluate the degradation rate, cement with a size of ɸ 6 × 7 mm was soaked in phosphate buffer solution (PBS, pH = 7.4) with a surface area to solution volume ratio of 0.1 cm^2^/mL [41,42]. Then, the cement and degradation solution were placed in a shaking water bath (37 °C, 80 rpm). The pH value of each solution was measured using a pH meter (PHS-3C, Shanghai Instrument Electric Science Instrument, Shanghai, China) after soaking for 1, 3, 7, 14, 28, and 42 days, and the PBS solution was replaced every seven days. The degradation rate of the bone cement was determined by measuring the mass change at different time points. Specifically, the cement column was removed from the degradation solution at different incubation periods and then dried in a vacuum oven at 60 °C for 24 h. The weight loss rate was then calculated using Equation (1)

(1) $$Weight\;loss\left(\%\right)=(1-\frac{m_{t}}{m_{0}})\times 100\%$$

where *m*_0_ denotes the initial mass of the cement, and *m_t_* denotes the mass of the cement after degradation [43]. Each test was performed in triplicate.

### 2.6. Release of ATP

For the in vitro ATP release experiments, cement with the same dimension as used for the weight loss tests was soaked in PBS solution (pH = 7.4) with a surface area and solution volume ratio of 0.1 cm^2^/mL [44]. Then, a centrifuge tube containing the cement and buffer solution was placed in a shaking water bath (37 °C, 80 rpm). Meanwhile, 3 mL of supernatant was removed after 4, 8, 12, 24, 48, 72, 96, and 120 h, and 3 mL of fresh PBS solution was replenished. To quantify the content of ATP, the standard curve of ATP was characterized by ultraviolet–visible (UV–Vis) spectrophotometry. The supernatant was filtered using a 0.22 μm filter membrane; the absorbance was measured by a UV–Vis spectrophotometer (UV-1800, Shimadzu, Kyoto, Japan) at 259 nm, and the concentration of ATP at different time points was determined by the standard curve of ATP. Three parallel samples were used for each treatment group. The percentage of ATP released was plotted versus the immersion time, and the release mechanism of ATP was simulated by the Ritger–Peppas model, given by Equation (2)

(2) $$y=M_{t}/M_{\infty }=kt^{n}$$

where *y* and *M_t_*/*M_∞_* is the cumulative released fraction at time *t*, *k* is the kinetic constant, and *n* is the characteristic parameter that indicates the transport mechanism in the release process [43].

### 2.7. Cell Proliferation and Fluorescence Staining

Mouse bone marrow mesenchymal stem cells (mMSCs; BNCC Biotechnology, Xinyang, China) were cultured in a complete culture medium (CCM) and then placed in an incubator (CO_2_ 5%, 37 °C, humidity 100%). The extract solutions of the specimen were diluted to concentrations of 200, 50, and 25 mg/mL. The extract solutions of ATP and ATP/CH were also diluted to different concentrations, as shown in Table 2.

The cell proliferation of the mMSCs was quantitatively assessed using the Cell Counting Kit-8 (CCK-8; Keygen Biotech, Nanjing, China). In short, 100 μL of the cell suspension (1 × 10^4^ cells per mL) were seeded in a 96-well plate, and the CCM was replaced with the extract after one day. Testing was carried out on days 1, 3, and 5, and the optical density (OD) value at 450 nm was measured using a microplate reader (Multiskan FC, Thermo Fisher Scientific, Shanghai, China). Six parallel samples were used for each treatment group. Finally, the relative survival rate (RSR) was calculated using Equation (3).

(3) $$RSR=({OD}_{extract}/{OD}_{CCM})\times 100\%$$

In the cell staining experiment, 1000 μL of the cell suspension (2 × 10^4^ cells per mL) were seeded in a 24-well plate, and the CCM was replaced with the extract after one day. On day 3, the cells were fixed with 2.5% glutaric dialdehyde solution, and 0.5 vol% Triton^TM^ X-100 was added to obtain transparency. The cytoplasm was stained using rhodamine phalloidin (Shanghai Yisheng Biotechnology, Shanghai, China), and the nucleus was stained using 4′,6-diamidino-2-phenylindole dihydrochloride (DAPI; Shanghai Yisheng Biotechnology, Shanghai, China), following a previously described protocol [45]. Finally, the cell morphology was examined using a Nikon SMZ800 stereomicroscope equipped with a Nikon camera. Three parallel samples were used for each treatment group.

### 2.8. Osteogenic Differentiation

Qualitative and quantitative tests of the calcium nodules were performed to evaluate the osteogenic induction ability. Firstly, 500 μL of the cell suspension (5 × 10^4^ cells per mL) were seeded in 48-well plates. Two days later, CCM was replaced with the osteogenic induction medium (iM) and osteogenic induction extracts, and the cells were cultured for 21 days. iM and the osteogenic induction extracts contained 0.1 μM dexamethasone, 10 mM β-sodium glycerophosphate, and 0.05 mM ascorbic acid. After 21 days of cell culture, the cells were fixed with a 2.5% glutaric dialdehyde solution. Then, the cells were stained with Alizarin Red dye, and the calcium nodules were photographed using a digital camera and an inverted microscope (ECLIPSE Ti, Nikon, Tokyo, Japan). Finally, the calcium nodules were dissolved in a 10% (*w*/*v*) cetylpyridinium chloride solution and, then at 620 nm, were measured using a microplate reader. Five parallel samples were used for each treatment group.

### 2.9. Statistical Methods

The data were expressed as mean ± standard deviation (SD) for the parallel samples. The statistical analyses between different groups were determined using a student’s *t*-test (* *p* < 0.05, ** *p* < 0.01, and *** *p* < 0.001).

## 3. Results and Discussion

### 3.1. Characterization

**Figure 2:**
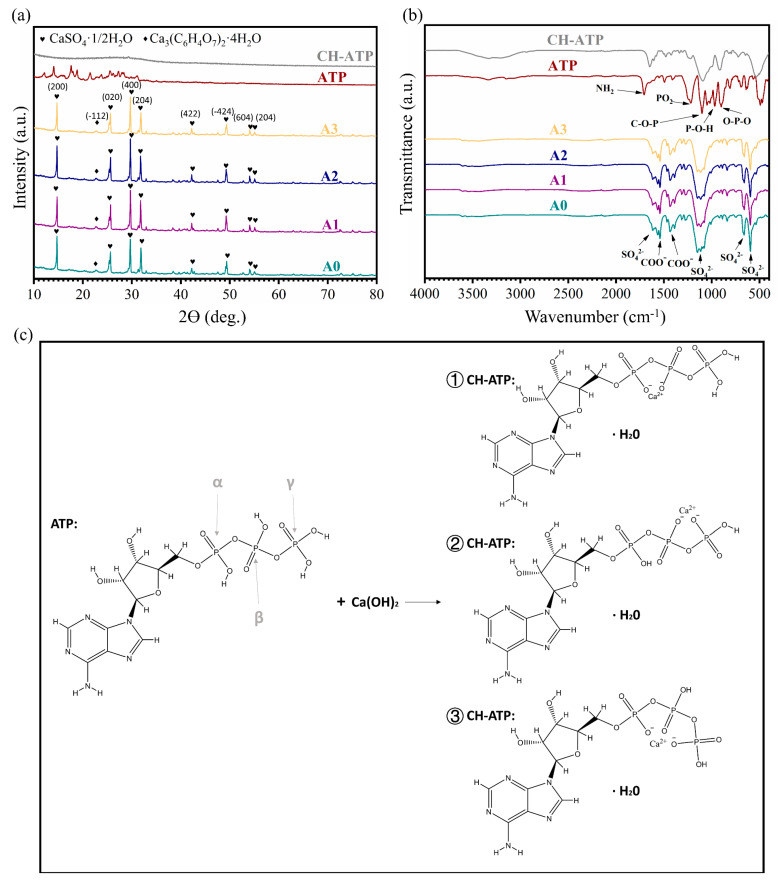
(**a**) XRD patterns and (**b**) FT-IR spectrum of ATP/CSH/CCT (A0, A1, A2, and A3) cement, ATP, and CH-ATP. (**c**) The reaction mechanism of the transition from ATP into CH-ATP.

The XRD patterns of the ATP/CSH/CCT (A0, A1, A2, and A3) types of cement shown in Figure 2a are similar, with discernible main diffraction peaks of CaSO_4_·1/2H_2_O and Ca_3_(C_6_H_5_O_7_)_2_·4H_2_O for samples A0, A1, A2, and A3, whereas the CaSO_4_·2H_2_O phase is not discernible. The hydration of CSD cement is attributed to a phase transition from CaSO_4_·1/2H_2_O to CaSO_4_·2H_2_O and is widely recognized as a rapid curing mechanism for CSD cement [46]. However, when ATP/CSH/CCT reacts with water, a large amount of Ca_3_(C_6_H_5_O_7_)_2_·4H_2_O crystals nucleate and grow on the surfaces of CaSO_4_·1/2H_2_O particles, blocking CSH hydration to some extent [47]. Therefore, for the composite cement, the major diffraction peaks of CaSO_4_·1/2H_2_O but not CaSO_4_·2H_2_O are visible. The characteristic diffraction peaks of ATP are not clearly visible in the XRD patterns of samples A1, A2, and A3, mainly because of their low ATP content.

Figure 2b shows the results obtained from FT-IR analysis of the different samples. For A0, A1, A2 and A3, the characteristic absorption bands of SO_4_^2−^ (~1622, ~1117, ~670, and ~601 cm^−1^) are attributed to CSH. Moreover, the characteristic absorption bands of COO^−^ (~1540 and ~1436 cm^−1^) are attributed to CCT. The characteristic absorption bands of ATP are not clearly visible in the FT-IR patterns of samples A1, A2, and A3, mainly because of their low ATP content.

Since ATP is a minor component in the composite cement and it is used with CH in liquid form, ATP and CH-ATP are detected as well. As shown in Figure 2a, ATP exhibits a crystalline phase, whereas CH-ATP exhibits a typical amorphous state. The FT-IR spectra of ATP and CH-ATP show significant differences. For ATP, the peak at 1708 cm^−1^ is assigned to the pyrimidine exocyclic NH_2_ deformation mode, and 1215 cm^−1^ is assigned to the PO_2_ anti-symmetric vibration. The C-O-P band occurs at 1103 cm^−1^, 966 cm^−1^ is assigned to a P-O-H stretching vibration, and 904 cm^−1^ is assigned to the O-P-O stretching band [48,49]. Compared with ATP, the peaks in CH-ATP shifted or disappeared. The peak at 1708 cm^−1^ shifted to 1643 cm^−1^ because of the molecular association in the CH and ATP mixture process. The P-O-H peak at 966 cm^−1^ disappeared, meaning all three phosphate groups were deprotonated. The absorption peak of the O-P-O bond at ~904 cm^−1^ in ATP shifted to ~917 cm^−1^ because of the strong, attractive force of Ca^2+^. Furthermore, the absorption peak of the C-O-P bond at ~1104 cm^−1^ shifted to ~1096 cm^−1^ because of the Ca^2+^ addition. In addition, the absorption band at 3100~3350 cm^−1^ is broader and stronger in CH-ATP, implying the presence of H_2_O molecules. These infrared data suggest the coexistence of α, β-, β, γ-, and α, γ-bidentates whereas the three phosphate groups (α, β, and γ) are the coordination sites of Ca^2+^ to ATP (Figure 2c).

**Figure 3:**
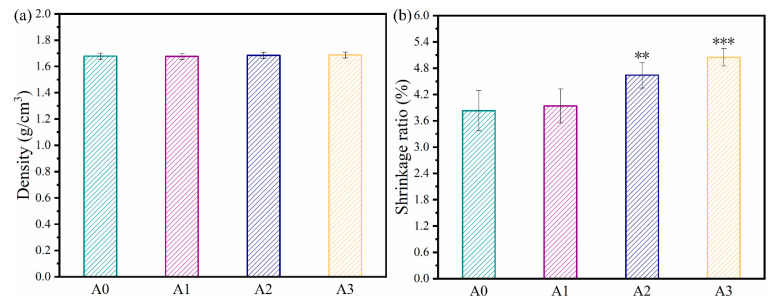
(**a**) Densities of ATP/CSH/CCT (A0, A1, A2, and A3) cement after curing in the air for three days. (**b**) Diameter shrinkage ratios of the ATP/CSH/CCT (A0, A1, A2, and A3) cement in comparison with the mold (** *p* < 0.01 and *** *p* < 0.001 compared with A0).

The densities of A0, A1, A2, and A3 are ~1.68, ~1.68, ~1.68, and ~1.69 g/cm^3^, respectively (Figure 3a). The results indicated that incorporating ATP into the cement did not significantly alter its density. The diameter shrinkage ratios of A0, A1, A2, and A3 after sitting in the air for three days are ~3.8%, ~3.9%, ~4.6%, and ~5.1%, respectively (Figure 3b). Evidently, with increased ATP loading, the diameter shrinkage ratios of bone cement samples gradually increase. This can be explained as follows: as the acid-base neutralization reaction between ATP and CH could lead to the production of water, the actual liquid-to-solid ratio of the bone cement paste will increase with an increasing ATP content. Hence, with increased ATP loading, the water loss rate of the cement will increase after three days of curing. As a result, higher ATP content resulted in a higher diameter shrinkage ratio.

**Figure 4:**
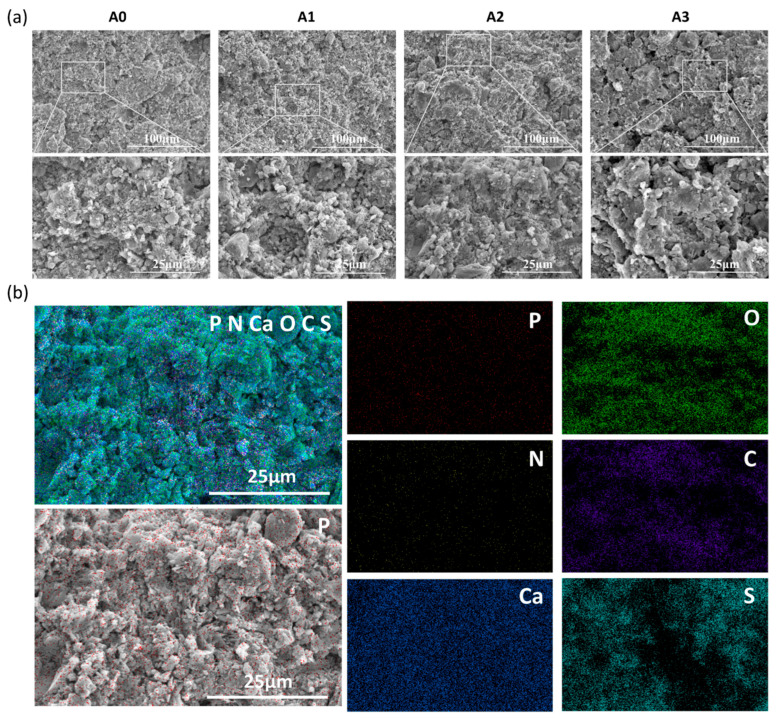
(**a**) The morphology of the cross-sections for ATP/CSH/CCT (A0, A1, A2, and A3) cement. (**b**) EDS mapping of A2 (including the distribution of P, N, Ca, O, C, and S).

The morphologies of the cross-sections of the ATP/CSH/CCT samples are shown in Figure 4a. The composite cement loaded with ATP has larger granules than the composite cement without ATP. Moreover, the interior microstructure of A3 is not as compact as the bone cement samples with low ATP loading (A0, A1, A2), as ATP might weaken the bonding between CSH and CCT. To further explore the distribution of ATP in the cement, the mapping of A2 is shown in Figure 4b. The P element is uniformly distributed in the cement. Because ATP is the sole source of the P element, this finding indicates a uniform distribution of ATP in the cement.

### 3.2. Compressive Strength

**Figure 5:**
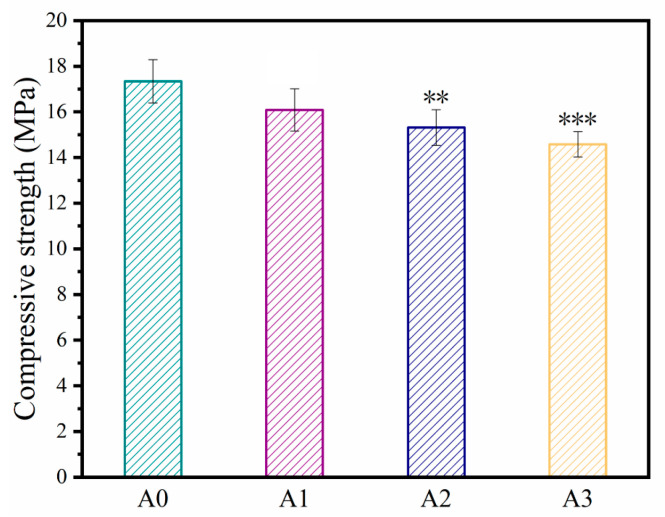
Compressive strengths of ATP/CSH/CCT (A0, A1, A2, and A3) cement (** *p* < 0.01 and *** *p* < 0.001 compared with the A0 sample).

The compressive strengths of bone cement samples with different ATP contents are shown in Figure 5. Evidently, with increased ATP loading, the compressive strength of the cement gradually decreases. The compressive strengths of A0, A1, A2, and A3 are ~17.3, ~16.1, ~15.3, and ~14.6 MPa, respectively. The compressive strength of human cancellous bone ranges between 5 and 10 MPa [50,51]. The compressive strengths of the fabricated samples were higher than 14 MPa, sufficient to meet the mechanical requirements of human cancellous bone. The compressive strength of the composite cement gradually decreases with increasing ATP loading ratio, probably because ATP combines with Ca^2+^ and weakens the absorbability of C_6_H_5_O_7_^3−^ to Ca^2+^.

### 3.3. In Vitro Degradation

**Figure 6:**
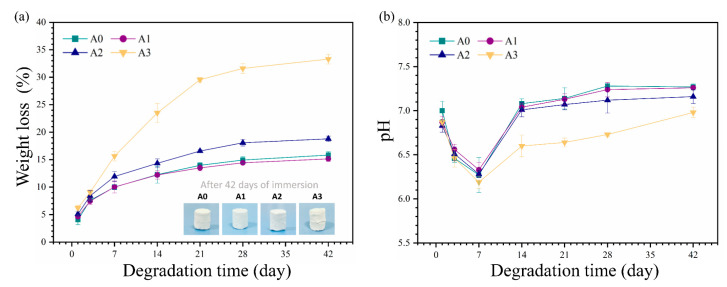
(**a**) Weight loss rates of ATP/CSH/CCT (A0, A1, A2, and A3) cement. Insets show the digital photos of each cement after soaking in PBS for 42 days. (**b**) pH variation of the degradation solution for ATP/CSH/CCT (A0, A1, A2, and A3) cement.

For ideal bone regeneration, a suitable degradation rate is required to replace the bone cement with new bone tissue gradually. The weight loss rates of the cement samples in vitro are shown in Figure 6a. The weight loss rate of the cement continuously increased with time but stagnated after soaking for 21 days. The weight loss rates of A0, A1, A2, and A3 were 14.0%, 13.5%, 16.6%, and 29.6%, respectively. After soaking for 42 days, the weight loss rates of A0, A1, A2, and A3 were 15.83%, 15.14%, 18.8%, and 33.3%, respectively. The mass loss is due to the dissolution of calcium sulfate hemihydrate and calcium citrate tetrahydrate. The chemical schemes of dissolution are given in Equations (4) and (5) [52,53]. Furthermore, a comparison of the degradation of the composite cement samples showed that higher ATP content resulted in a faster degradation rate, as ATP might weaken the bonding between CCT and CSH.

(4) $${CaSO}_{4}(s)⇌{Ca}^{2+}(aq)+{SO}_{4}^{2-}(aq)\;K_{sp}\;=\;3.14\;\times \;10^{-5}$$

(5) $${Ca}_{3}{\left(C_{6}H_{5}O_{7}\right)}_{2}(s)⇌3{Ca}^{2+}(aq)+{2C_{6}H_{5}O_{7}}^{3-}(aq)\;K_{sp}\;=\;8.7\;\times \;10^{-17}$$

Figure 6b shows the pH variation of the composite cement suspensions during the degradation of the cement samples in PBS. During the first seven days, the pH values of the suspensions (A0, A1, and A2) presented a downward trend and reached the lowest value on the 7th day. The pH of the suspensions (A0, A1, and A2) gradually stabilized to approximately 7.2. However, the pH values of the A3 cement suspensions were lower, causing a higher loss rate.

**Figure 7:**
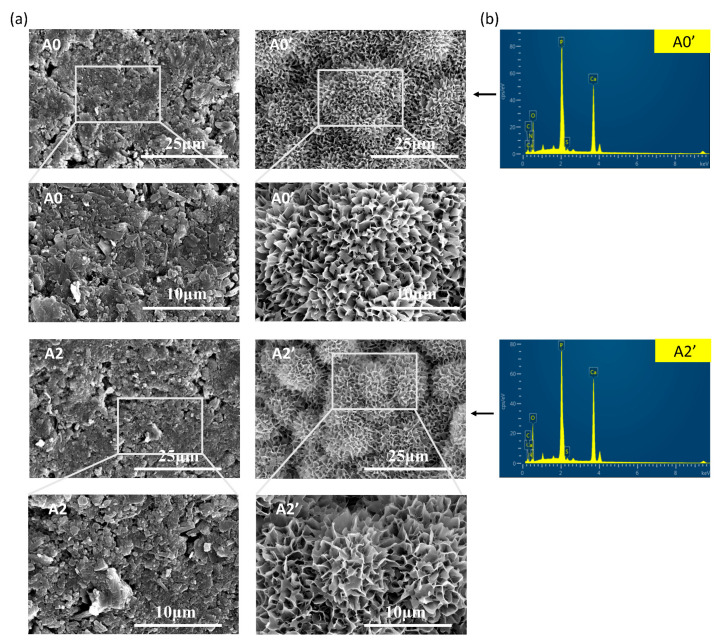
(**a**) SEM micrographs of the cement surfaces before (A0 and A2) and after (A0′ and A2′) soaking in PBS for 21 days. (**b**) EDS spectra for A0′ and A2′.

Theoretically, bone cement should continuously degrade because its components are degradable. Therefore, the samples were immersed in PBS for 21 days and examined by SEM, as shown in Figure 7a. Before soaking, the composite cement possessed a relatively compact surface structure with some micropores. Following soaking, the original granules disappeared, and the typical flower-like spherulites of the Ca-P layer were formed on the surface of the composite cement. The EDS spectra for ATP/CSH/CCT (A0, A2) composite cement samples after soaking in PBS for 21 days are shown in Figure 7b. Additionally, the EDS data are shown in Table 3, indicating that the cement surfaces were covered by apatite, preventing degradation.

### 3.4. In Vitro Release of ATP

**Figure 8:**
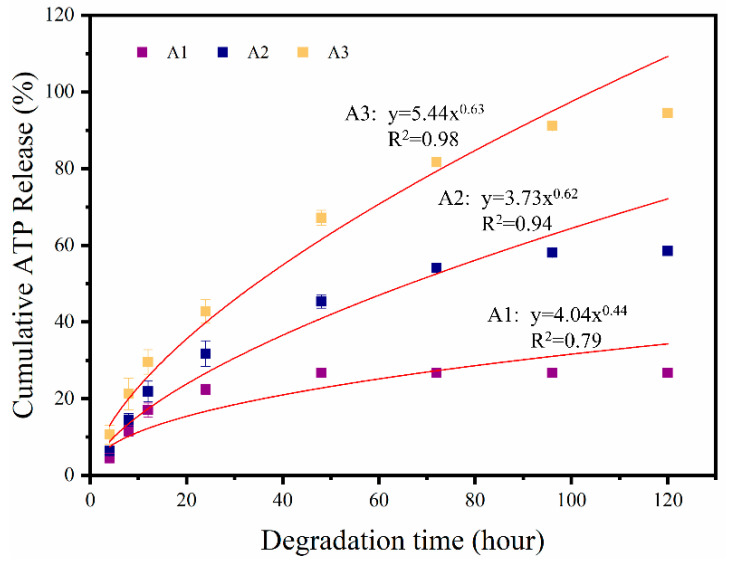
ATP cumulative release from ATP/CSH/CCT (A1, A2, and A3) cements in PBS.

The ATP release behavior from ATP/CSH/CCT (A1, A2, and A3) cement is presented in Figure 8. The simulation formulas of the release profiles are listed above the line. The cumulative release of ATP from the cement remained constant after initially increasing. With the gradual release of ATP, the cumulative release rates of ATP were approximately 26.74%, 58.6%, and 94.5% for A1, A2, and A3 after 120 h, respectively. The release mechanics of ATP from ATP/CSH/CCT were simulated by the Ritger–Peppas model; the corresponding formulas are shown in Figure 7. The parameter n characterizes the release mechanism, and the *n* values for A2 and A3 are 0.63 and 0.62, respectively, indicating that the release mechanism of ATP/CSH/CCT (A2 and A3) follows a non-Fickian diffusion mechanism (0.45 < *n* < 0.89). Therefore, the release of ATP from ATP/CSH/CCT relies on its diffusion and degradation of bone cement. However, the *n* value for A1 is 0.44, indicating that the release mechanism of A1 follows a Fickian diffusion mechanism (*n* < 0.45). Therefore, the release of A1 solely relies on its diffusion. As the concentration of the ATP in A1 is the lowest among the composites, the concentration gradient and the driving force for diffusion are small, resulting in a low release rate.

### 3.5. Cell Proliferation

Generally, it is useful to use cell culture experiments to evaluate the in vitro biocompatibility of materials. Figure 8 shows the results of the cell culture evaluation for the cytocompatibility of the composite cement and ATP. To determine the effect of the ATP and ATP/CH extracts on the composite cement, they were evaluated following the same procedure. Figure 8a demonstrates that the cytocompatibility of ATP and ATP/CH is very similar and beneficial for cell proliferation. Furthermore, C2 and D2 showed the best cell activity among all the extracts. The cell proliferation in the extracts of the composite cement was better than in the control sample, except for the 200 mg/mL extracts. Notably, the ATP-doped cement exhibited better cell proliferation than A0 on the first day. At an ATP/CSH/CCT extract concentration of 50 mg/mL, mMSCs showed good activity on bone cement (Figure 8b). Comparing the cell proliferation rates of the composite cement samples (50 mg/mL) revealed that the A2 extracts yielded the best viability.

**Figure 9:**
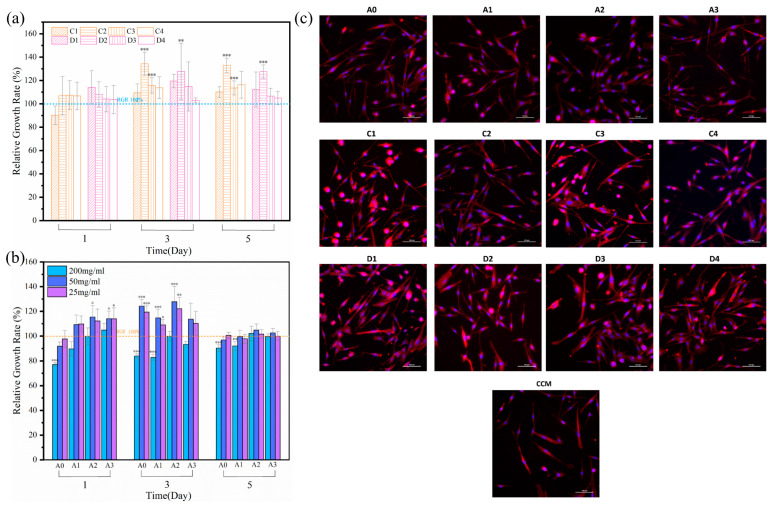
(**a**) Relative cell growth rate cultured with ATP and ATP/CH extracts detected by a CCK-8 proliferation assay at different times. (**b**) Relative cell growth rate cultured with ATP/CSH/CCT extracts was detected by a CCK-8 proliferation assay at different times. (**c**) FIM photos of mMSCs cultured in CCM; ATP, ATP/CH, and ATP/CSH/CCT extracts after three days of incubation (scale bar, 100 μm. * *p* < 0.05, ** *p* < 0.01, and *** *p* < 0.001 compared with CCM).

Figure 9a shows that both the ATP and ATP/CH extracts (C, D) have excellent cytocompatibility. Figure 9b shows that the composite cement extracts (50 mg/mL) exhibit a more significant stimulatory effect on mMSCs proliferation than CCM, indicating that the composite cement is non-cytotoxic. Furthermore, the A2 composite cement (50 mg/mL) shows the best combination of ATP and CSH/CCT for stimulating cell proliferation, as appropriate concentrations of ATP and dissolution products, including Ca^2+^ and C_6_H_5_O_7_^3−^, released from the composite cement are favorable for stimulating cell proliferation [54,55,56].

Because the CCK-8 results showed that the cell survival rates of the ATP, ATP/CH, and ATP/CSH/CCT extracts (50 mg/mL) were suitable, these extracts were selected for fluorescence staining studies. As shown in Figure 9c, the majority of the mMSCs cells had a spindle shape, with distinct pseudopodia and good cell morphology. Additionally, compared with the cells in CCM, a greater number of cells were observed in the ATP, ATP/CH, and ATP/CSH/CCT extracts (50 mg/mL).

### 3.6. Cell Differentiation

**Figure 10:**
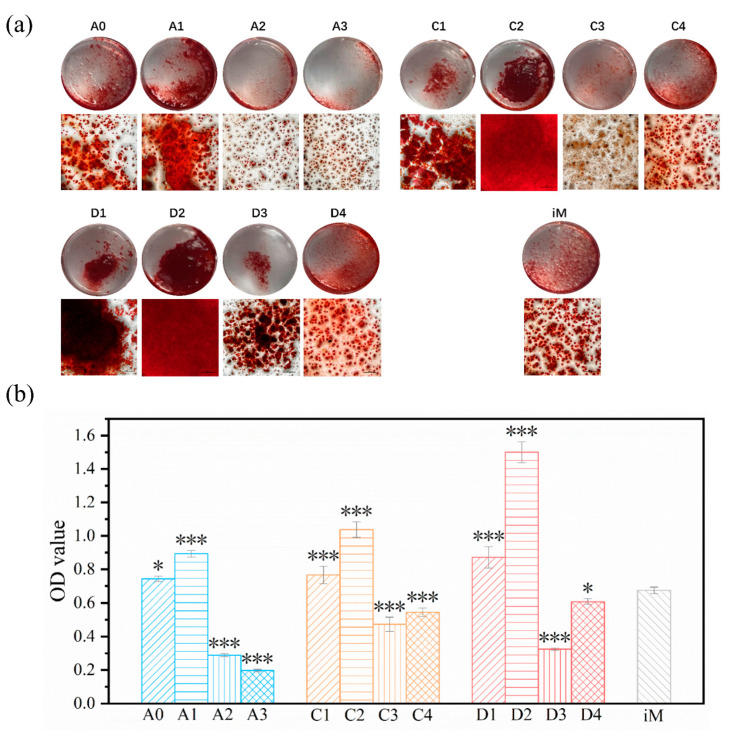
(**a**) Alizarin red S staining photos of mMSCs cells cultured in iM and osteogenic induction extracts for 21 days (scale bar, 500 μm). (**b**) The OD value of mMSCs cells stained with alizarin red S after 21 days of incubation (* *p* < 0.05 and *** *p* < 0.001 compared with iM).

Because the proliferation results showed that the ATP, ATP/CH, and ATP/CSH/CCT extracts (50 mg/mL) were cytocompatible, these extracts were selected for differentiation studies. Calcium nodules are late markers of mMSCs differentiation into osteoblasts. The calcium nodules secreted during the differentiation of mMSCs were stained with alizarin red S and then subjected to semi-quantitative analysis. The degree of extracellular matrix calcification of mMSCs cultured with iM and osteogenic induction extracts for 21 d is shown in Figure 10a,b. After 21 days, there were more calcium nodules in A1 than in iM and A0. In contrast, the calcium nodules in A2 and A3 were less than those in iM, indicating that the composite cement loaded with a certain concentration of ATP (A1) was conducive to differentiating mMSCs into osteoblasts. Furthermore, the calcium nodules in C1 and C2 were higher than in iM. In comparison, the calcium nodules in C3 and C4 were lower than those in iM, indicating that a specific concentration range of ATP could promote the differentiation of mMSCs into osteoblasts. Figure 10 also shows that the cytoactivity of ATP and ATP/CH is very similar.

Previous studies have shown that ATP can differentiate BMSCs into osteoblasts [26,57]. According to the quantitative analysis results (Figure 9b), the calcium nodules in C1 and C2 are more than those in the control group, indicating that a certain concentration range of ATP (312.5–1250 μg/mL) promotes mMSCs differentiation. Additionally, the A1 composite cement (50 mg/mL) exhibits the best ATP and CSH/CCT combination for stimulating mMSCs differentiation. This is because appropriate concentrations of ATP and Ca^2+^ released from composite cement are conducive to differentiating mMSCs into osteoblasts [58].

## 4. Conclusions

In this study, ATP/CSH/CCT cement was successfully prepared for the first time. The density of ATP/CSH/CCT is approximately 1.68 g/cm^3^. The compressive strength and weight loss rates were investigated, and the resulting materials presented appropriate compressive strength (14.6–17.3 MPa) and weight loss rates (15.14–33.3%), meeting the clinical requirements of degradable bone cement. The cement induced the deposition of a Ca-P layer in PBS. Cytocompatibility studies showed that this type of bone cement promotes the proliferation and differentiation of mMSCs. Considering the ideal degradability and cytoactivity, the prepared ATP/CSH/CCT cement is a promising bone substitute for implantation.

## Figures and Tables

**Table 1:** Compositions of ATP/ CSH/CCT cements.

Cement Label	Solid Phase		Liquid Phase			Liquid–Solid Ratio
	CSH (g)	CC (g)	0.15% CH (mL)	CMCS (mg)	ATP (mg)	(mL/g)
A0	6	4	3	200	0	0.3
A1	6	4	3	200	10	0.3
A2	6	4	3	200	50	0.3
A3	6	4	3	200	100	0.3

**Table 2:** Concentration of ATP and ATP/CH extract solutions.

	ATP				ATP/CH			
Extract Solutions	C1	C2	C3	C4	D1	D2	D3	D4
ATP (μg/mL)	1250	312.5	78.13	19.53	1250	312.5	78.13	19.53
CH (μg/mL)	0	0	0	0	112.5	28.13	7.03	1.76

**Table 3:** The EDS about the atomic percentage on the cement surface and the Ca/P atomic ratios.

	P (%)	N (%)	Ca (%)	O (%)	C (%)	S (%)	Ca/P
A0	16.39	0	24.99	42.35	16.14	0.13	1.52
A2	15.99	0	25.94	45.56	12.27	0.24	1.62

## Data Availability

Not applicable.
